# A Resonant Pressure Microsensor with Temperature Compensation Method Based on Differential Outputs and a Temperature Sensor

**DOI:** 10.3390/mi11111022

**Published:** 2020-11-21

**Authors:** Chao Xiang, Yulan Lu, Pengcheng Yan, Jian Chen, Junbo Wang, Deyong Chen

**Affiliations:** 1 State Key Laboratory of Transducer Technology, Aerospace Information Research Institute, Chinese Academy of Sciences, Beijing 100190, China; xiangchao115@mails.ucas.ac.cn (C.X.); luyulan15@mails.ucas.ac.cn (Y.L.); yanpengcheng17@mails.ucas.ac.cn (P.Y.); chenjian@mail.ie.ac.cn (J.C.); 2 School of Electronic, Electrical and Communication Engineering, University of Chinese Academy of Sciences, Beijing 100049, China

**Keywords:** resonant pressure microsensor, silicon-on-glass cap, temperature compensation, polynomial fitting

## Abstract

This paper presents the analysis and characterization of a resonant pressure microsensor, which employs a temperature compensation method based on differential outputs and a temperature sensor. Leveraging a silicon-on-insulator (SOI) wafer, this microsensor mainly consists of a pressure-sensitive diagram and two resonant beams (electromagnetic driving and electromagnetic induction) to produce a differential output. The resonators were vacuum packaged with a silicon-on-glass (SOG) cap using anodic bonding and the wire interconnection was realized by sputtering an Au film on highly topographic surfaces using a hard mask. After the fabrication of the resonant pressure microsensor, systematic experiments demonstrated that the pressure sensitivity of the presented microsensor was about 0.33 kPa/Hz. Utilizing the differential frequency of the two resonators and the signal from a temperature sensor to replace the two-frequency signals by polynomial fitting, the temperature compensation method based on differential outputs aims to increase the surface fitting accuracy of these microsensors which have turnover points. Employing the proposed compensation approach in this study, the errors were less than 0.02% FS of the full pressure scale (a temperature range of −40 to 85 °C and a pressure range of 200 kPa to 2000 kPa).

## 1. Introduction

As a common physical quantity, pressure has been widely used in many areas, such as industrial control, meteorological monitoring, oil exploration and aerospace investigation [1,2,3,4]. Compared with other pressure sensors, resonant pressure sensors are featured with high performances in linearity, resolution, stability and accuracy. Furthermore, the output of resonant pressure sensors is a quasi-digital signal, which has high robustness [5,6,7,8].

Due to temperature-property mismatches among micromachined materials, resonant pressure microsensors always suffer from the key issue of resonant frequency drift due to temperature variations regardless of exciting modes and fabrication steps [9,10,11]. Temperature drift of the output frequency brings large errors and severely compromises the accuracy of the sensors. Hence, resonant pressure sensors have to employ temperature compensation methods [12,13].

Temperature compensations can be classified into two types, hardware compensations (inclusion of additional temperature-sensitive elements) and software compensations. In 2005, Chen et al. reported a resonant pressure sensor with temperature compensation, where three doubly clamped beams were used for pressure measurements and a pair of cantilevers were used for temperature measurements [14]. Nevertheless, the fabrication of this resonant pressure sensor was full of challenges. Then, a dual-beam silicon-based resonant pressure microsensor was developed by Tang et al. in 2011 [15], where one working beam and one compensating beam isolated from pressure variations were used with the difference of the dual beams’ resonant frequencies as the indicator of temperature. However, this approach suffered from limited accuracy since after temperature compensation, the maximal residual errors were decreased to 1.38% FS in a temperature range of −40 to 60 °C.

Software compensations were conducted with the help of an external temperature sensor and mathematic algorithms [16], which can be classified into four main types: Least square [17,18], polynomial fitting [19,20], support vector machine [21] and back-propagation (BP) neural network [22,23]. The approach of least square is widely used to describe the linear relationship between variables and is compatible with curve fitting. However, when the relationship between the two variables is nonlinear, there is a compromised accuracy for the compensation. Polynomial fitting is a common method which combines the advantages of least square and the superiority of polynomial expansion. With the help of Taylor’s expansion, formulas of variables can be obtained with corresponding coefficients obtained based on least square. Nevertheless, if the functions of the variables are not monotonous, the compensation accuracy may also be compromised. As one of key algorithms in machine learning, support vector machine itself leads to limited compensation accuracy. When the sum value of error squares in the target function is added, the precision of temperature compensation can be effectively improved. Yet, the corresponding formula cannot be obtained in this way, leading to limited applications. Neural network is a large-scale nonlinear dynamic system, capable of fitting any nonlinear function at any accuracy. As a widely used neural network model, BP neural network is a unidirectional multi-layered feed-forward network, based on the principle of stochastic approximation, which, however, suffers from complicated calculation processes.

Different with the aforementioned two types of temperature compensations, self-temperature compensation does not need additional temperature sensors or temperature sensitive elements, which relies on self-temperature properties of microsensors [24]. More specifically, a silicon resonant pressure sensor employing self-temperature compensation was introduced by Jan in 1996 where a least-square fit of calibration data based on measurements of resonant frequencies for two different oscillation modes of a single resonator was used [25]. However, the disadvantages of this method were large residual errors, over ±0.1% FS, and inherently slow readout during measurements, due to switching times between two oscillation modes. In order to realize high accurate measurements and avoid the steps of switching modes, previously, our group presented a resonant pressure microsensor capable of self-temperature compensation based on two resonant frequencies derived from two doubly-clamped resonant beams in 2015, reporting the maximal errors of less than 0.01% of the full pressure scale, temperature range of −40 °C to 70 °C and pressure range of 50 kPa to 110 kPa [26]. Employing self-temperature compensation, our group presented a microsensor which worked in a pressure range of 10 kPa to 150 kPa and a temperature range of −35 °C to 85 °C, in 2018 [27]. Nevertheless, this approach suffered from a key limitation that if the measurement ranges of temperature and pressure were expanded (temperature range of −40 °C to 85 °C and pressure range of 200 kPa to 2000 kPa), a turnover temperature point appeared as a local zero temperature coefficient of frequency [28], leading to large fitting errors.

For the sake of meeting the measurement requirements of wide temperature ranges and high-pressure measurement accuracy, this study presented a resonant pressure sensor where temperature compensation based on the combination of differential output of two resonators and external temperature sensor was demonstrated. Employing this method, the measurement errors of pressure were less than 0.02% FS under the pressure range of 0–2 MPa and the temperature range of −40 °C to 85 °C. The paper (1) analyzed the working principle of the resonant pressure sensor, (2) simulated the presented microsensor by finite element analysis (FEA), (3) conducted the fabrication of the resonant pressure microsensor, (4) put forward the temperature compensation method based on differential outputs and a temperature sensor and (5) validated the advantage of the presented temperature compensation method with experiments.

## 2. Theoretical Analysis of the Microsensors

### 2.1. Microsensor’s Structure and Its Output Signal Analysis

**Figure 1:**
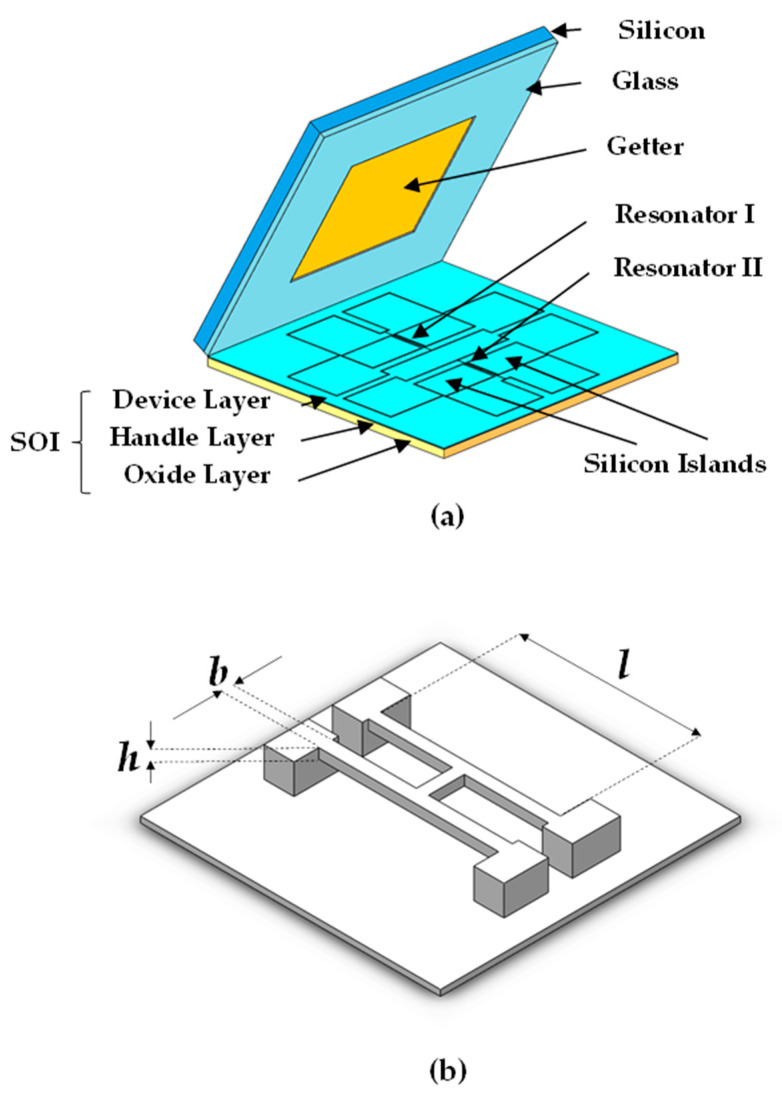
Schematic diagram of the resonant pressure sensor with an SOG cap: (**a**) the sensor consists of an SOI part (a pressure sensitive diaphragm, two H-shaped beams, four silicon islands and eight through silicon vias) and a SOG cap with getter and (**b**) structural parameter of the resonant beam.

The developed resonant pressure microsensor which employed electromagnetic driving and induction included a silicon-on-insulator (SOI) layer and a silicon-on-glass (SOG) cap (see Figure 1a). In the SOI layer, double “H” beams (length: 1400 μm, width: 18 μm and thickness: 40 μm) were fixed on a pressure-sensitive diaphragm (length: 10200 μm, width: 10200 μm and thickness: 300 μm) through the anchor. The pressure under measurement modulates axial stress of H beams, producing an increase and a decrease in the resonant frequencies of the resonator I and II, respectively, as a differential output. This differential design can improve the performance (e.g., sensitivity and linearity) of the presented resonant pressure microsensor [29]. Moreover, silicon islands placed on device layer of SOI were used to expand the pressure measurement range, for it equaled to increase the thickness of pressure sensitive diaphragm.

The structure of the “H” beam is showed in Figure 1b where *h* is the thickness of the “H” beam, *b* is the width of the “H” beam, *l* is the length of the “H” beam, *E* is the Young’s modulus of silicon and ρ is the density of silicon.

Assuming the surrounding temperature and the pressure is constant, the resonant frequency of the H-beam can be simplified according to the theory of elastic mechanics [30]:

(1) $$f_{0}=f(0)=\frac{4.73^{2}}{2\pi l^{2}}\sqrt{\frac{EI}{A\rho }}=1.028\sqrt{\frac{E}{\rho }}\cdot \frac{b}{l^{2}},$$

When the pressure changes, the axial stress appears and the intrinsic frequency changes, and the axial stress is the function of pressure. Therefore, this intrinsic frequency is the function of axial stress and pressure.

According to Equation (1), the resonant frequency of the beam depends on the length of the beam, the width of the beam, the density of silicon and the Young’s modulus of silicon, when the surrounding pressure is constant. Meanwhile, E,ρ,b,l are affected by temperature variations. Due to the coefficient of thermal expansion (CTE) mismatching between silicon and glass, the process of anodic bonding also brings residual stresses in the microsensor, which influence the intrinsic frequency of the resonant beam. These stresses are the function of temperature.

Assuming the pressure is constant, the basic resonant frequency of the resonant beam is the function of surrounding temperature. Hence, the resonant frequency of the resonant beam is the function of pressure and surrounding temperature.

### 2.2. FEA Simulations of the Sensor’s Characteristics

**Figure 2:**
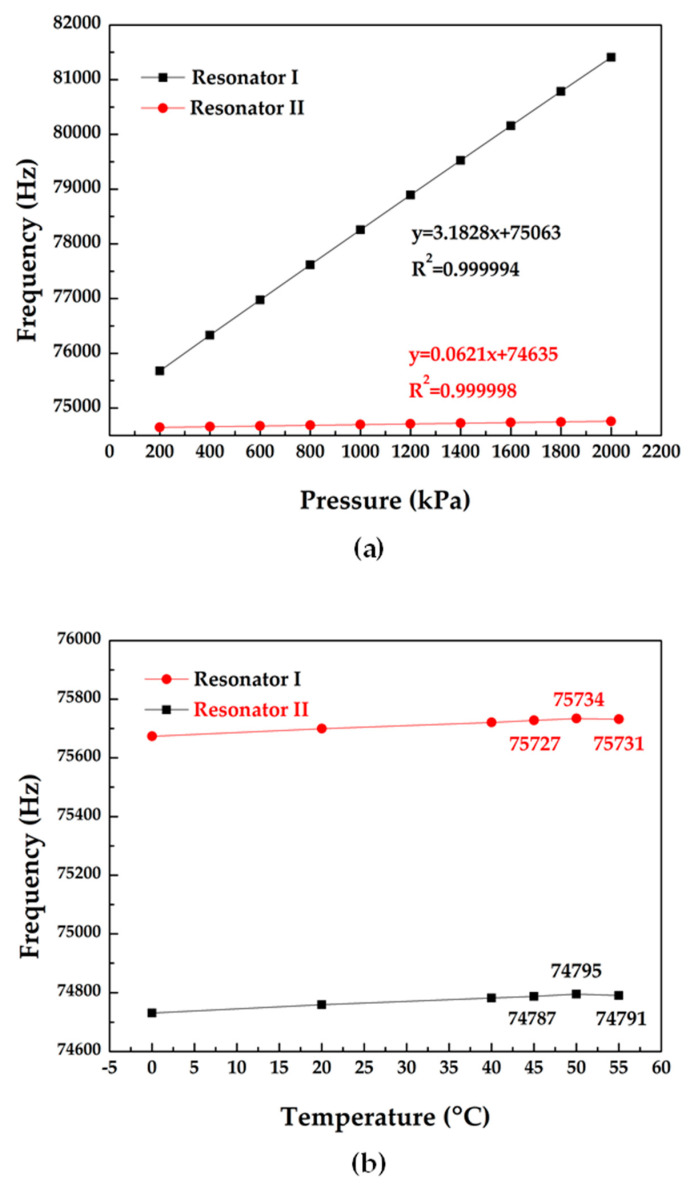
(**a**) the intrinsic frequency responses to applied pressure in the room temperature and (**b**) the intrinsic frequency responses to surrounding temperature at 200 kPa.

In order to analyze the frequency drift caused by temperature variations, FEA simulations based on ANSYS (Pittsburgh, PA, USA) were conducted to obtain the pressure and temperature properties of the sensor using the SOG cap (see Figure 2a,b). The dimension information of the microsensor model employing in FEA simulations are listed in Table 1.

The shifts of intrinsic frequencies responding to pressure variances and temperature variances were calculated based on modal in the toolbox. Meanwhile, for the purpose of meshing geometrical structures of the microsensors, tetrahedrons were used due to the geometry considerations. In order to realize high-precision simulations, the element size of the SOI part was set to 100 μm and the element size of the resonant beam was set to 5 μm. After the process of meshing, the number of elements was 447,661.

Furthermore, the relationship between applied pressure, surrounding temperature and the resonant frequencies was also obtained (see Figure 2a,b). According to Figure 2a, the pressure sensitivities of the two resonators were quantified as 3.18 Hz/kPa and 0.06 Hz/kPa in the room temperature. Figure 2b shows that the turnover temperature points appear in the range of 45–55 °C. This simulation results suggested that the temperature-frequency curves of the two resonators are not monotonous in the working temperature range of −40–85 °C.

## 3. Temperature Compensation Method Based on Differential Outputs and a Temperature Sensor

### 3.1. Self-Temperature Compensation and Its Limitations

According to the analysis in Section 2, the output resonant frequency of the microsensors can be expressed as the function of pressure and surrounding temperature. This function is quite complex. However, with the help of mathematical translation, pressure can be expressed as the function of frequency and surrounding temperature.

If the relationship between frequency and temperature is monotonous, temperature can also be expressed as the function of frequency and pressure. Therefore, pressure can be expressed as the function of the two resonant frequencies. Though, this function cannot be described easily, it can still be obtained by polynomial fitting of the calibrations data [26]. After that, pressure can be expressed as follows:

(2) $$\begin{array}{l} P=a_{0}+a_{1}f_{1}^{5}+a_{2}f_{1}^{4}f_{2}+a_{3}f_{1}^{4}+a_{4}f_{1}^{3}f_{2}^{2}+a_{5}f_{1}^{3}f_{2}+a_{6}f_{1}^{3}+a_{7}f_{1}^{2}f_{2}^{3}\\ +a_{8}f_{1}^{2}f_{2}^{2}+a_{9}f_{1}^{2}f_{2}+a_{10}f_{1}^{2}+a_{11}f_{1}f_{2}^{4}+a_{12}f_{1}f_{2}^{3}+a_{13}f_{1}f_{2}^{2}+a_{14}f_{1}f_{2}\\ +a_{15}f_{1}+a_{16}f_{2}^{5}+a_{17}f_{2}^{4}+a_{18}f_{2}^{3}+a_{19}f_{2}^{2}+a_{20}f_{2}+\varepsilon \end{array},$$

where *a*_0_, …, *a*_20_ are constant coefficients and ε is a higher-order infinitesimal.

In order to determine the coefficients in Equation (2), a calibration was conducted in the full pressure range from 200 kPa to 2000 kPa, and the temperature range from −40 °C to 85 °C. The specific pressure points for calibration were 200 kPa, 400 kPa, 600 kPa, 800 kPa, 1000 kPa, 1200 kPa, 1400 kPa, 1600 kPa, 1800 kPa and 2000 kPa while the specific temperature points were −40 °C, −30 °C, −20 °C, −10 °C, 0 °C, 20 °C, 40 °C, 60 °C, 80 °C and 85 °C. In the subsequent pressure measurements, the compensated pressure values were obtained by substituting the resonant frequencies of two resonators into the compensation function.

Nevertheless, the FEA simulations showed that the relationship between resonant frequencies of resonators and surrounding temperature is not monotonous in the temperature range of −40 to 85 °C (see Figure 2b). In this scenario, one value of frequency corresponded to two values of temperature. Therefore, the relationship between frequency and surrounding temperature is not a single-valued function, and the absolute errors of temperature measurements by self-temperature compensation increased dramatically, leading to the huge pressure measurement errors. That is the limitation of self-temperature compensation.

### 3.2. Working Principle of the Presented Temperature Compensation Method

In order to decrease the absolute errors of pressure measurement, an input which contains temperature information must be added. Moreover, according to the analysis in Section 3.1, differential outputs *f_d_* can also be expressed as the function of pressure and surrounding temperature.

Since the two resonators were placed in the same vacuum cavity, the stresses on the two resonators caused by the variation of materials’ properties were comparable. Thus, *f_d_* can also decrease the common-mode interferences well. Therefore, two frequencies can be replaced with differential outputs and a temperature signal to conduct temperature compensation.

And polynomial fitting of the calibrations data can also get the formula of pressure. Hence, pressure can be expressed:

(3) $$\begin{array}{l} P=a_{0}+a_{1}f_{d}^{5}+a_{2}f_{d}^{4}T+a_{3}f_{d}^{4}+a_{4}f_{d}^{3}T^{2}+a_{5}f_{d}^{3}T+a_{6}f_{d}^{3}+a_{7}f_{d}^{2}T^{3}\\ +a_{8}f_{d}^{2}T^{2}+a_{9}f_{d}^{2}T+a_{10}f_{d}^{2}+a_{11}f_{d}T^{4}+a_{12}f_{d}T^{3}+a_{13}f_{d}T^{2}+a_{14}f_{d}T\\ +a_{15}f_{d}+a_{16}T^{5}+a_{17}T^{4}+a_{18}T^{3}+a_{19}T^{2}+a_{20}T+\varepsilon \end{array},$$

For the sake of getting the temperature value, this paper utilized a temperature sensor to obtain accurate temperature values. During the processes of calibration, a temperature sensor was positioned near the resonant pressure microsensor.

The process of temperature compensation was based on three steps:

(1) Calibration: Place the resonant pressure sensor and the temperature sensor in a temperature chamber and set the temperature and pressure of the calibration point on a host computer. After the temperature and pressure reached the preset values and stabilized for a certain time, the frequency values of the double resonators were recorded by the frequency collection system and the value of the temperature sensor was measured by a multimeter synchronously. (2) Surface fitting: After the calculation of the differential frequencies obtained by calibration, the data was normalized at the same time with the values obtained from the temperature sensor. Then MATLAB based on polynomial fitting was conducted, obtaining the function of temperature compensation. (3) Pressure measurement: The acquisition system measured the frequency values of the two resonators, calculated the differential frequency and obtained the value of the temperature sensor. After data normalization, the processed data was introduced into the function of temperature compensation to obtain the pressure value.

### 3.3. Selection of External Temperature Sensor

According to the theory of temperature compensation, the proper selection of an external temperature sensor has a significant influence on the pressure measurement errors. A high-accuracy temperature sensor can be very expensive, while the compensation accuracy may decrease if the accuracy of the temperature sensor is too low. Therefore, it is necessary to properly select the external temperature sensor to find a balance between accuracy and cost.

So as to select a suitable temperature sensor meeting high accuracy measurement, several error fittings were conducted based on microsensor’s calibration data and four different temperature measurement accuracies to obtain their maximum measurement errors (see Table 2).

Table 2 shows the results of error fitting for (1) temperature measurement errors of ±5 °C with the maximum pressure measurement error of 364.29 Pa, over ±0.018% FS; (2) temperature measurement errors of ±1 °C with the maximum pressure measurement error of 217.74 Pa, higher than ±0.01% FS and (3) temperature measurement errors of ±0.01 °C with the maximum pressure measurement error of −195.54 Pa. To meet the accuracy requirement of pressure measurements, the accuracy of temperature sensors should be higher than ±0.01 °C. Since the temperature chamber’s control accuracy cannot be ignored and the size of the temperature sensor should be small enough to be positioned close to the pressure-sensitive microsensor, in this study, PT1000 was used as the temperature sensor featured with high accuracy in a temperature measurement less than ±0.01 °C from −40 °C to 85 °C [31].

## 4. Device Fabrication

The sensor’s fabrication which employed MEMS technology contained the fabrication of the SOI wafer and SOG cap. The fabrication processes of the SOI wafer began with a 4″ SOI wafer ((100) plane, <100> oriented, p-type, device layer of 40 μm, handle layer of 300 μm and oxide layer of 2 μm).

**Figure 3:**
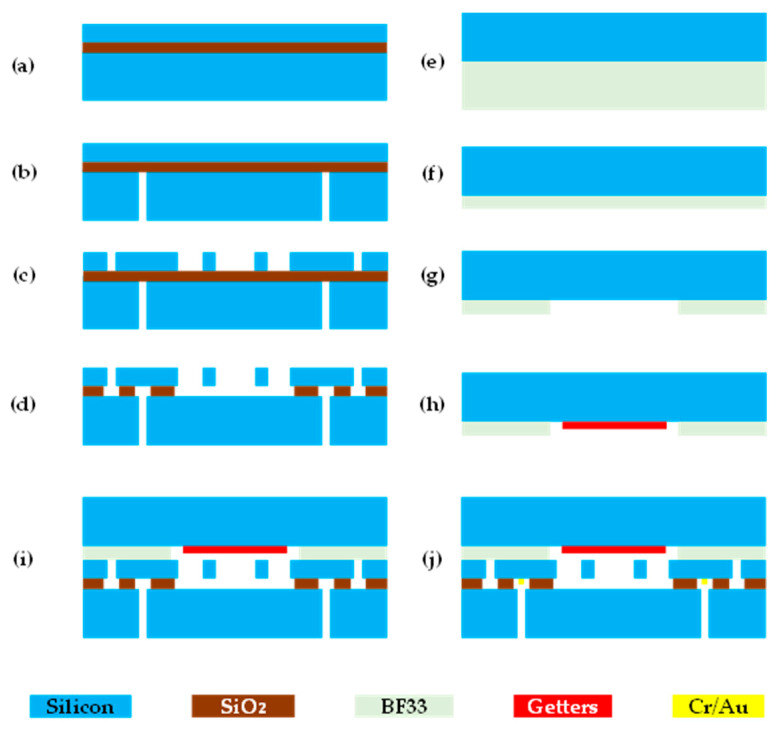
The fabrication processes of the resonant pressure micro sensor based on SOI-MEMS technologies: (**a**) cleaning the SOI wafer; (**b**) lithography and deep reactive ion etching (DRIE) for vias; (**c**) lithography and DRIE for resonators; (**d**) releasing of resonant beams; (**e**) Si-glass anodic bonding; (**f**) chemical mechanical planarization (CMP) of the bonding wafer; (**g**) lithography and etching for cavity; (**h**) sputtering the getter; (**i**) SOI-SOG anodic bonding and (**j**) sputtering metal electrodes.

The SOI wafer was thoroughly cleaned by a standard wafer cleaning process to meet the requirements of MEMS technologies (see Figure 3a). Then, the AZ4620 photoresist (AZ Electronics Materials, Somerville, MA, USA) of ~5 μm was spun and patterned on the handle layer, which functioned as the etching mask to defined via holes. The via holes were defined by deep reactive ion etching (DRIE) (see Figure 3b). After that, using patterned photoresist AZ4620 as a mask, the device layer was etched through by DRIE to make resonators (see Figure 3c). As the next step, the buried silicon oxide layer under the resonators was removed in HF vapor (see Figure 3d).

The SOG cap’s fabrication started with anodic bonding between one 4-inch 500 μm borosilicate glass wafer and one 4-inch 500 μm P-type <100> silicon wafer, under a voltage of 1000 V, 0.1 Pa and 350 °C, respectively (see Figure 3e). Then, the chemical mechanical planarization (CMP) was employed to decrease the thickness of glass layer to ~100 μm (see Figure 3f). After that, the bonding wafer was cleaned to remove particulates. As the next step, the cavity for vacuum package was made by etching about 40 μm depth (see Figure 3g). After thoroughly cleaning the wafer, getter was sputtered on the cavity to absorb gas releasing during next anodic bonding (see Figure 3h).

After finishing the fabrication of the SOI wafer and SOG wafer, vacuum packaging was conducted by anodic bonding between the two wafers under a voltage of 350 V, 0.1 Pa and 350 °C, respectively (see Figure 3i). Finally, Cr/Au films were sputtered on the through-silicon via to prepare for electrical connections employing a hard mask (see Figure 3j).

**Figure 4:**
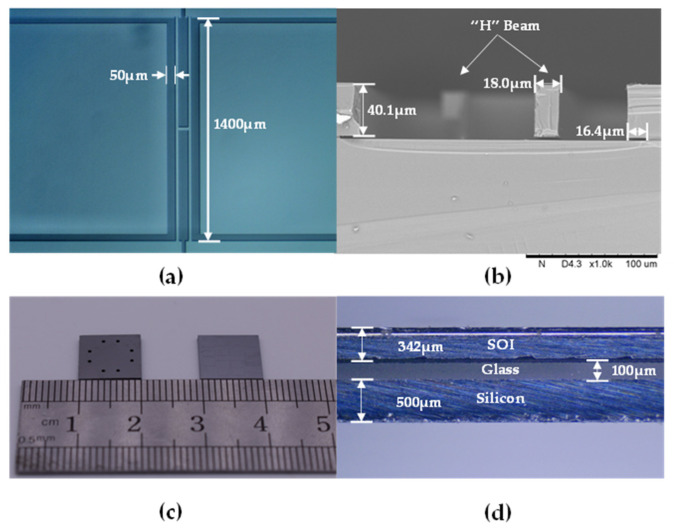
The fabrication results: (**a**) top view of the “H” resonant beam; (**b**) SEM image of the resonant beam; (**c**) front/back views of the microsensor chips and (**d**) side view of the microsensor chip.

The fabrication results were shown in Figure 4, including the top view of the “H” resonant beam (see Figure 4a), the scanning electron microscopy (SEM) image of the resonant beam (see Figure 4b), the front/back views of the microsensor chips after dicing (see Figure 4c), the side view of the microsensor chip (see Figure 4d).

## 5. Characterizations

For the sake of further validating the advantage of the temperature compensation method based on differential outputs and a temperature sensor, a closed-loop circuit was developed to obtain the stable intrinsic frequencies of the developed microsensor. A pressure calibrator (PPC4, FLUCK) and a temperature chamber (SU-242, ESPEC) were employed to control the pressure and surrounding temperature during the calibration processes. In this study, the developed microsensor was characterized within a pressure range from 200 kPa to 2 MPa and a temperature range from −40 to 85 °C.

**Figure 5:**
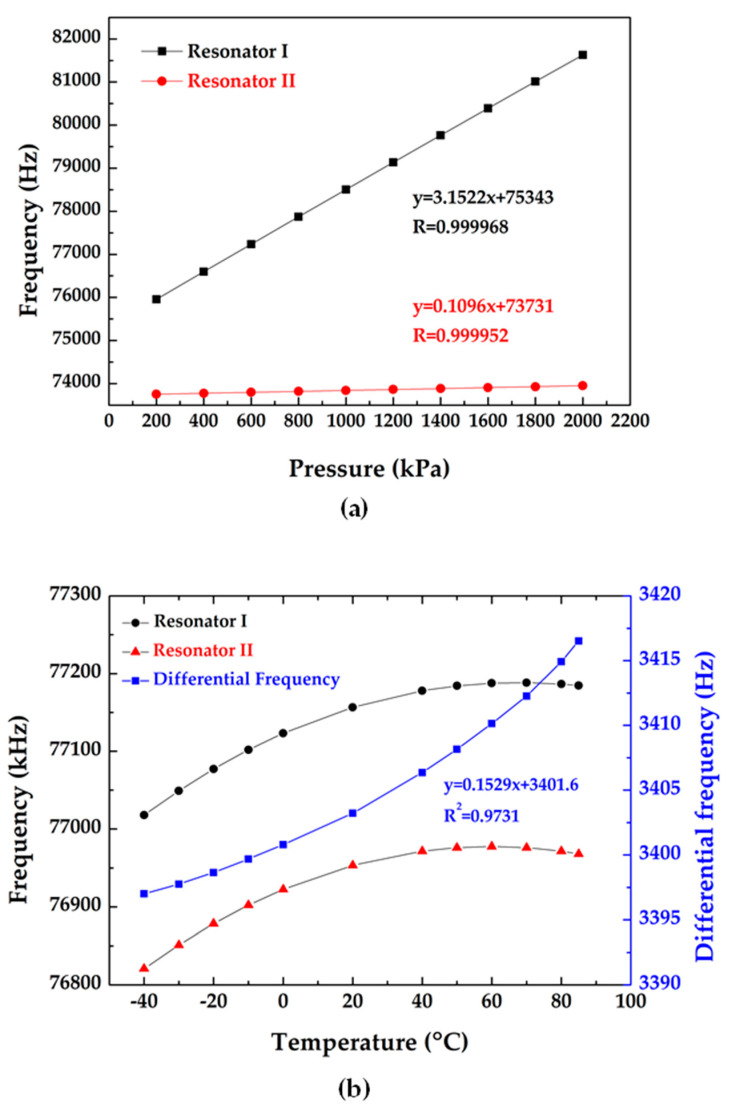
Experimental results: (**a**) the frequency response as functions of the applied pressures at 20 °C and (**b**) the frequency response as functions of the surrounding temperatures at 200 kPa.

The intrinsic frequencies of the microsensor in response to pressure variation at 20 °C were recorded (see Figure 5a). In accordance with Figure 5a, the pressure sensitivities were quantified as 3.15 Hz/kPa with a correlation coefficient of 0.999968 (Resonator I) and 0.11 Hz/kPa with a correlation coefficient of 0.999952 (Resonator II). Plus, Figure 5b showed the turnover temperature points existed in the range from 40 to 70 °C.

**Figure 6:**
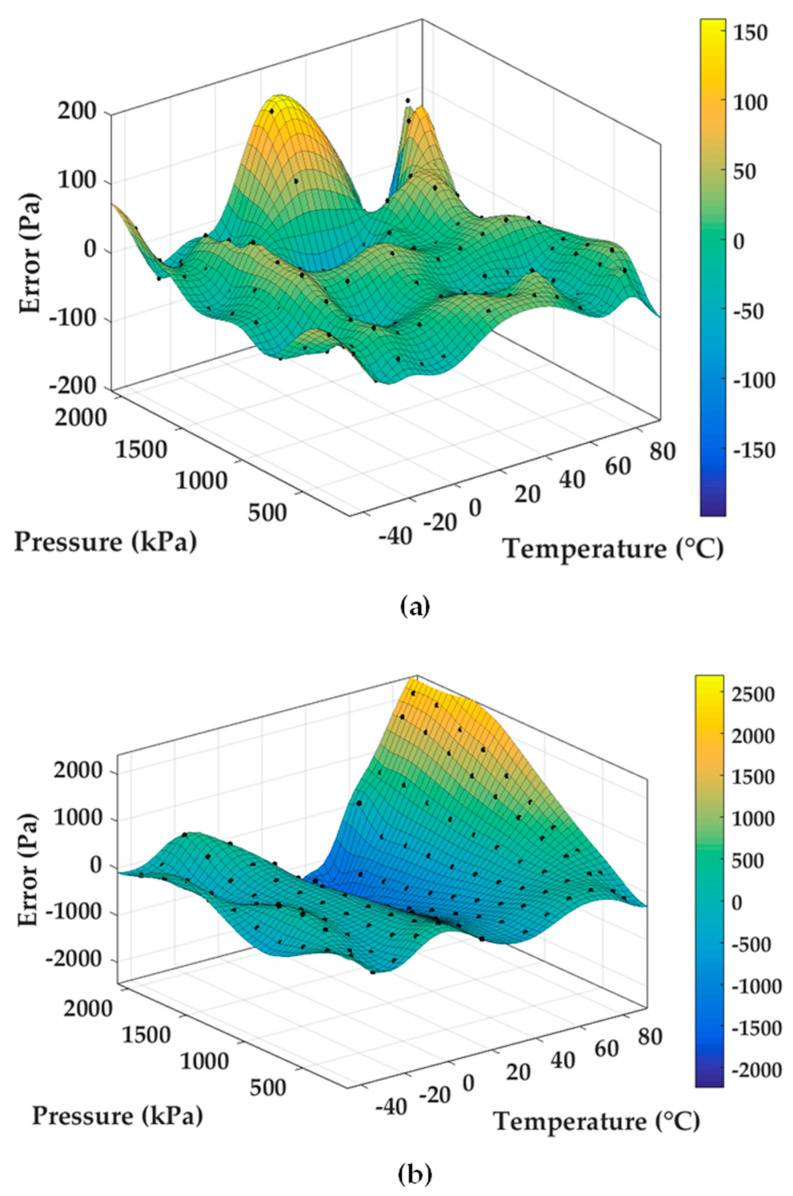
Experimental results: (**a**) pressure’s surface fitting of the temperature compensation method based on differential outputs and a temperature sensor applied on the developed sensor and (**b**) pressure’s surface fitting of the self-temperature compensation method applied on the developed sensor.

To compare the accuracy of the presented temperature compensation method with the self-temperature compensation method, the two output frequencies of the microsensor and the resistance of PT1000 were collected in calibration. After processing the data in MATLAB, the results of surface fitting were shown in Figure 6a,b. According to Figure 6a,b, the fitting errors based on the presented temperature compensation method were less than 200 Pa while the fitting errors based on self-temperature compensation method were more than 2000 Pa, under the pressure range of 200 kPa to 2 MPa. These results indicated that the temperature compensation method based on differential outputs and a temperature sensor can obtain measurements with higher accuracies, compared with self-temperature compensation method.

**Figure 7:**
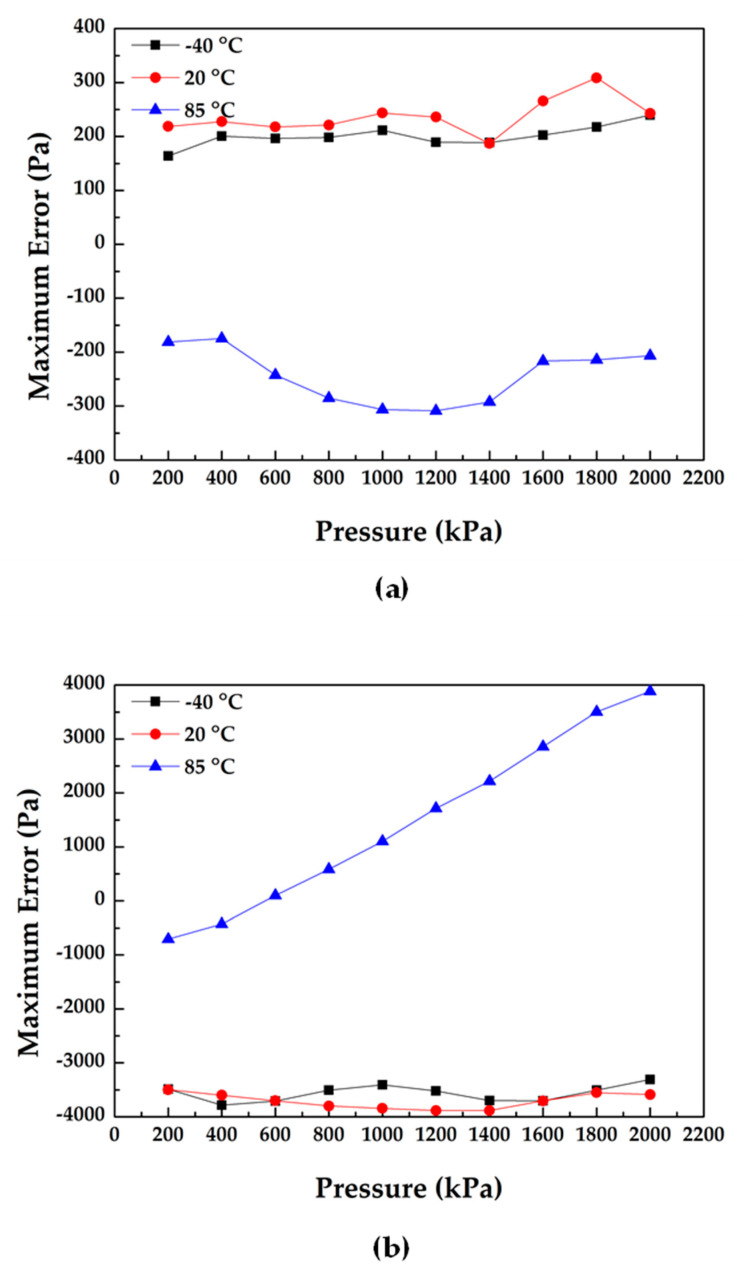
Experimental results: (**a**) the maximum measurement errors of the temperature compensation method based on differential outputs and a temperature sensor in three cycles and three different temperatures and (**b**) the maximum measurement errors of the self-temperature compensation method in three cycles and three different temperatures.

Moreover, the experiments also suggested that temperature compensation method based on differential outputs and a temperature sensor obtained low maximum measurement errors of about ±0.02% FS under the pressure range of 200 kPa to 2 MPa in three cycles while, self-temperature compensation method obtained big maximum measurement errors of ±0.2% FS, under the same pressure range (see Figure 7a,b). These results further validated the superiority of the approach of temperature compensation method based on differential outputs and a temperature sensor.

## 6. Conclusions

A resonant pressure microsensor employing temperature compensation method based on differential outputs was presented in this paper. The microsensor was fabricated based on conventional fabrication processes. According to the experimental results, the pressure sensitivities of the presented microsensor’s two resonators were 3.15 Hz/kPa and 0.11 Hz/kPa at 20 °C, with linear correlation coefficients of 0.999968 and 0.999952. These results matched the simulations well, which validated the design. With the help of the high accurate temperature sensor PT1000, the system can obtain the temperature value surrounding the resonators. In addition, employing temperature compensation method based on differential outputs and a temperature sensor, the maximum fitting errors were decreased from over ±2000 Pa to less than ±200 Pa under the pressure range of 0–2 MPa in a temperature range of −40 °C to 85 °C. Further characterizations showed that the measurement errors were less than ±0.02% FS in three cycles and under the pressure range of 0–2 MPa in −40 °C, 20 °C, 85 °C.

## Figures and Tables

**Table 1:** Dimension information of the microsensor model employing in finite element analysis (FEA) simulations.

Part	Length/μm	Width/μm	Thickness/μm	Depth/μm
Resonant beam	1400	18	40	-
Device layer of SOI	10,200	10,200	40	-
Handle layer of SOI	10,200	10,200	300	-
Oxide layer of SOI	10,200	10,200	2	-
Cavity of SOG cap	5160	5160	-	40
Glass layer of SOG	10,200	10,200	100	-
Silicon layer of SOG	10,200	10,200	500	-

**Table 2:** Error fitting results with different temperature measurement accuracies.

Temperature Measurement Accuracy	Maximum Pressure Measurement Error
±5 °C	364.29 Pa
±1 °C	217.74 Pa
±0.1 °C	198.39 Pa
±0.01 °C	195.55 Pa
